# Performance of large language models (GPT-5.2, Gemini 3 Pro, Claude Sonnet 4.6 and Grok 4.1) on the Fellowship of The Royal College of Surgeons Urology Part A examination

**DOI:** 10.1136/bmjopen-2025-108775

**Published:** 2026-05-13

**Authors:** Abdul Rhaman Kafagi, Nazih Atassi, Abdul-Hadi Kafagi

**Affiliations:** 1 Faculty of Biology Medicine and Health, The University of Manchester, Manchester, England, UK; 2 Department of Urology, Wythenshawe Hospital, Manchester University Hospitals NHS Foundation Trust, Manchester, England, UK

**Keywords:** Artificial Intelligence, UROLOGY, SURGERY, MEDICAL EDUCATION & TRAINING, Machine Learning

## Abstract

**Abstract:**

**Objectives:**

To assess and compare the performance of four contemporary frontier large language models (LLMs)—GPT-5.2 (OpenAI), Gemini 3 Pro (Google DeepMind), Claude Sonnet 4.6 (Anthropic) and Grok 4.1 (xAI)—on a simulated Fellowship of The Royal College of Surgeons Urology (FRCS(Urol)) Part A examination, evaluating overall accuracy, subspecialty-level performance, output consistency and response time.

**Design:**

Controlled comparative evaluation study using a standardised simulation framework with repeated independent testing runs per model.

**Setting:**

All models were accessed via their respective consumer-facing interfaces. No clinical setting or patient data were involved. Testing was conducted under uniform conditions with conversational memory disabled across all sessions.

**Participants:**

Four large language models were evaluated. No human participants were involved. Models were selected to represent the current frontier of publicly accessible LLMs from four distinct commercial developers. No models were excluded following selection.

**Interventions:**

Each model was presented with 240 FRCS (Urol) Part A single best answer questions, mapped to the Joint Committee on Intercollegiate Examinations' Urology Syllabus Blueprint (2023). A standardised prompt was delivered at the start of each session. Each model completed five independent examination runs. No fine-tuning or system-level modification was applied to any model.

**Primary and secondary outcome measures:**

The primary outcome was overall examination accuracy for each model, benchmarked against an indicative pass threshold for the FRCS (Urol) Part A examination. Secondary outcomes were performance across 18 individual urology subspecialty topics; response time reported as mean total and per-question elapsed time; and consistency of performance quantified by SD and 95% CIs derived from a sequential Monte Carlo sampling procedure. All outcomes were prospectively planned and fully measured as specified.

**Results:**

Three of four models exceeded the indicative 74% pass threshold: Gemini 3 Pro (82.4%±0.9%; 95% CI 81.3 to 83.6%), Claude Sonnet 4.6 (79.3%±1.1%; 95% CI 77.9 to 80.6%) and GPT-5.2 (76.1%±2.4%; 95% CI 73.1 to 79.1%). Grok 4.1 failed (70.4%±0.6%; 95% CI 69.6 to 71.2%), with its entire CI below 74%. All models completed the assessment in under 3 min. Strong performance was observed in research methodology (90–98%) and andrology (92–98%), with the weakest results in paediatric urology (38.7–54.7%) and testicular cancer (48.2–67.3%). Substantial within-model output instability was identified across several domains, most notably GPT-5.2 in female urology (SD±22.8%) and anatomy (SD±14.2%).

**Conclusions:**

Three of four frontier LLMs achieved scores consistent with passing the FRCS (Urol) Part A examination, representing a substantial advance since ChatGPT-3.5. Aggregate accuracy alone, however, obscures important subspecialty weaknesses and output instability. LLMs should be regarded as adjunctive revision aids rather than authoritative knowledge sources and always used alongside expert-led teaching. Future work should evaluate performance on Part B and viva-style assessments.

**STRENGTHS AND LIMITATIONS OF THIS STUDY**

- A standardised simulation framework, including identical prompt delivery, disabled conversational memory and deactivated internet access across all sessions, ensured methodological consistency and comparability across all four models.
- A sequential Monte Carlo sampling procedure was used to construct 95% CIs with a fixed precision threshold, providing a principled and adaptive approach to quantifying performance uncertainty rather than relying on an arbitrary number of replicates.
- Models were evaluated through consumer-facing interfaces rather than controlled API access, introducing potential variability attributable to server load and interface-level processing that cannot be fully accounted for.
- Performance on Part B written papers, viva examinations and clinical scenario assessments, which evaluate applied reasoning and professional judgement, was not assessed.

##  Introduction

Artificial intelligence (AI) has undergone rapid development in recent years, particularly in the field of natural language processing (NLP). Among the most notable advancements is the emergence of large language models (LLMs), which use deep learning techniques to generate human-like text based on vast datasets. Since the landmark release of ChatGPT by OpenAI in November 2022, the field has progressed at an extraordinary pace, with successive generations of LLMs demonstrating increasingly sophisticated capabilities in reasoning, contextual understanding and domain-specific knowledge application.^1^ LLMs work by learning the likelihood of sequential word patterns from training on large text datasets, enabling them to simulate conversations, answer questions and perform language-related tasks with increasing sophistication. Their widespread adoption in various industries, including customer service, education and healthcare, has prompted investigations into their potential for supporting complex professional domains such as medicine and surgery.

These models have demonstrated exceptional performance on general language tasks and standardised professional examinations such as the USMLE, MCAT and American Board examinations; however, their capabilities within specialised clinical domains, such as surgical subspecialties, remain less well characterised.^2^,^4^ A recent study by Desouky *et al* evaluated ChatGPT-3.5’s performance on the FRCS Urology exam, reporting a failure with a score of 35%.^5^ Subsequent generations of LLMs have since demonstrated marked improvements in performance, with GPT-4 surpassing its predecessor through enhanced reasoning, creativity and multimodal capabilities. The current landscape now encompasses a diverse ecosystem of frontier models from competing developers, including GPT-5.2 (OpenAI), Gemini 3 Pro (Google DeepMind), Claude Sonnet 4.6 (Anthropic) and Grok 4.1 (xAI), each employing distinct architectural approaches and training methodologies.^6^,^10^ Comparative evaluation of these models across high-stakes professional assessments is therefore essential to characterise their relative strengths and limitations.

Urology training demands a strong foundation of medical knowledge, clinical reasoning and surgical judgement, rigorously assessed by the Fellow of the Royal College of Surgeons (FRCS) Urology Part A examination. This high-stakes exam evaluates candidates’ broad understanding of urological sciences and is a mandatory requirement to obtain a Certificate of Completion of Training (CCT) in the UK. This study aims to assess and compare the performance of four contemporary frontier LLMs: GPT-5.2 (OpenAI), Gemini 3 Pro (Google DeepMind), Claude Sonnet 4.6 (Anthropic) and Grok 4.1 (xAI) on a curated set of FRCS Urology Part A examination questions.

By examining accuracy, subspecialty-level reasoning and response characteristics, we seek to explore the potential role of these cutting-edge models as supplementary tools in urology education, exam preparation and knowledge dissemination. Understanding the comparative strengths and limitations of these models within a specialised surgical context will provide valuable insights for their integration into medical training and continuous professional development.

## Methods

This study is reported in accordance with the STROBE (Strengthening the Reporting of Observational Studies in Epidemiology) guidelines (online supplemental file 1).

The primary outcome was overall examination accuracy for each model, benchmarked against the published FRCS (Urol) Part A pass mark to determine simulated pass or fail performance. Secondary outcomes included: performance across individual urology subspecialty topics as mapped to the Joint Committee on Intercollegiate Examinations' Urology Syllabus Blueprint (2023); response time per model, reported as mean total and per-question elapsed time across repeated independent runs; and consistency of performance.^11^

### Examination design and question selection

To assess the ability of selected LLMs to pass the Urology FRCS Urol Part A exam, we conducted a study using a series of 240 FRCS Part A questions. To replicate exam conditions, we divided the questions into two mock examinations. Each mock exam comprised 120 questions, encompassing both Paper 1 and Paper 2 components. By including both types of questions in our study, we aimed to replicate the comprehensive nature of the FRCS (Urol) Part A exam and evaluate their performance across different question formats and complexities. Questions were selected and mapped to the Joint Committee on Intercollegiate Examinations’ Urology Syllabus Blueprint (2023).^11^

### Model selection and access

All models (GPT-5.2 (OpenAI), Gemini 3 Pro (Google DeepMind), Claude Sonnet 4.6 (Anthropic) and Grok 4.1 (xAI)) were accessed via their respective consumer-facing interfaces. No fine-tuning or system-level modification was applied to any model prior to testing.

### Simulation framework

To ensure reproducibility and minimise instruction-induced variability, all models were tested under a standardised simulation framework. Each model was presented with an identical prompt at the start of every session:

“*This session will simulate the FRCS (Urol) Part A examination. You will be presented with multiple single best answer questions covering the urology syllabus. For each question, select the single best answer and return only the corresponding letter. Do not provide explanations unless specifically requested. Please confirm readiness*.”

Each examination run was conducted in a new independent session with conversational memory disabled, ensuring no influence from prior interactions. Image-based questions were presented using high-resolution figures extracted directly from the source question bank to maintain uniform visual input across all models. Questions were submitted sequentially within each session, and responses were marked against an official answer key. Accuracy was recorded at both the individual topic level and as an overall total score.

### Statistical analysis

Per-topic and overall scores were aggregated across repeated runs for each model. To quantify uncertainty in performance estimates, a sequential Monte Carlo sampling procedure was used to construct 95% CIs.^12^,^14^ Sampling continued until the half-width of the 95% CI was less than 2.5% of the point estimate, ensuring consistent precision across all models without requiring an arbitrarily fixed number of replicates. CIs were computed empirically from the resulting score distributions. Data were analysed using Stata and tabulated in Microsoft Excel. The published pass mark for the FRCS (Urol) Part A examination was used as the benchmark threshold for determining simulated pass or fail performance.

To evaluate model performance, accuracy rate (A) was calculated as the percentage of correctly answered questions.^15^ It is defined mathematically as:


$$A=\frac{N_{correct}}{N_{total}}\times 100\%$$

where Ncorrect is the number of questions answered correctly by a given model, and Ntotal is the total number of questions evaluated.

### Response time measurement

Response time was defined as the elapsed interval from submission of each question to receipt of the single-letter answer, encompassing model inference, server processing and network latency. Each examination run was timed using automated timestamps, and total elapsed time was divided by the number of questions to derive an average per-question response time. Timing was repeated across five independent runs per model.

### Patient and public involvement

Patients and/or the public were not involved in the design, or conduct, or reporting, or dissemination plans of this research.

## Results

### Time

The response times of the four AI models differed substantially (table 1). ChatGPT had the longest mean total time at 187.6±48.4 s (95% CI 127.5 to 247.7 s), corresponding to 0.78±0.20 s per question. Grok required 76.4±15.1 s in total (0.32±0.06 s per question; 95% CI 57.7 to 95.1 s). Claude had a mean total time of 64.6±31.7 s (0.27±0.13 s per question; 95% CI 25.2 to 104.0 s), while Gemini was the fastest, responding in 59.2±8.8 s (0.25±0.04 s per question; 95% CI 48.3 to 70.1 s).

**Table 1:** Mean total response times, time per question, SD and 95% CIs for four AI models (n=5)

Model	Mean time (s)	Time per question (s)	SD (s)	SD per question (s)	95% CI (s)	95% CI per question (s)
ChatGPT	187.6	0.782	48.42	0.202	127.5 to 247.7	0.531 to 1.032
Gemini	59.2	0.247	8.79	0.037	48.3 to 70.1	0.201 to 0.292
Grok	76.4	0.318	15.06	0.063	57.7 to 95.1	0.241 to 0.396
Claude	64.6	0.269	31.74	0.132	25.2 to 104.0	0.105 to 0.433

### Scores

**Figure 1:**
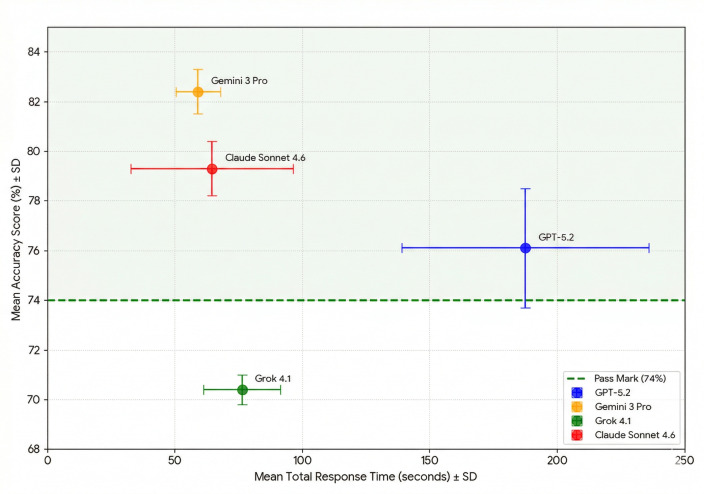
Comparison of exam performance across four AI models (n=5 per model). Box plots represent the score distribution, with individual data points overlaid. The vertical dotted green line indicates the passing threshold of 74%.

The mean total scores for the four models were as follows (mean±SD, 95% CI, n=5): ChatGPT 76.1%±2.4%, 73.1–79.1%; Gemini 82.4%±0.9%, 81.3–83.6%; Grok 70.4%±0.6%, 69.6–71.2%; and Claude 79.3%±1.1%, 77.9–80.6% (table 2; figure 1)

**Table 2:** Comparison of mean total scores, SD and 95% CIs across four AI models (n=5)

Model	Mean (%)	SD (%)	n	95% CI (%)
ChatGPT	76.1	2.4	5	73.1 to 79.1
Gemini	82.4	0.9	5	81.3 to 83.6
Grok	70.4	0.6	5	69.6 to 71.2
Claude	79.3	1.1	5	77.9 to 80.6

### Efficiency matrix—accuracy against speed

**Figure 2:**
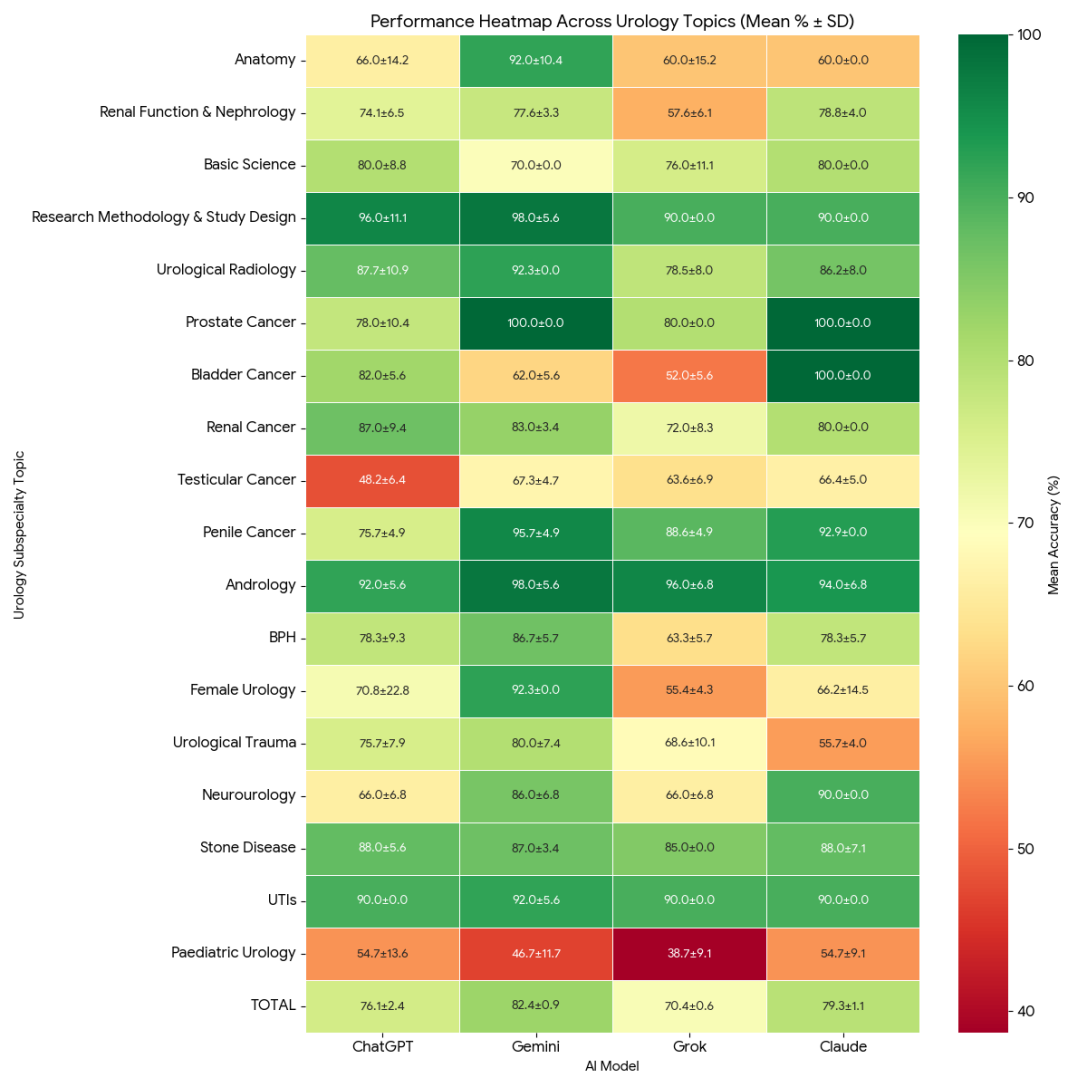
Accuracy versus efficiency and output instability.

The relationship between model accuracy and computational efficiency was evaluated through a bivariate efficiency matrix. Gemini 3 Pro emerged as the most efficient performer, occupying the optimal “top-left” quadrant of the matrix by achieving the highest mean accuracy (82.4 s) in the shortest mean response time (59.2). In contrast, GPT-5.2 demonstrated significant inefficiency, requiring the longest processing time (187.6 s) while exhibiting the greatest degree of output instability. While Claude Sonnet 4.6 maintained a balanced profile of high speed and accuracy, Grok 4.1 was the only model to fall consistently below the 74% pass mark, despite its competitive response latency (76.4%). Analysis of the 95% CIs and SD “target” regions further confirms that Gemini and Claude provide the most stable performance envelopes, whereas the high variability of GPT-5.2 makes its passing status sitting-dependent. (figure 2)

### Scores by specialty

**Figure 3:**
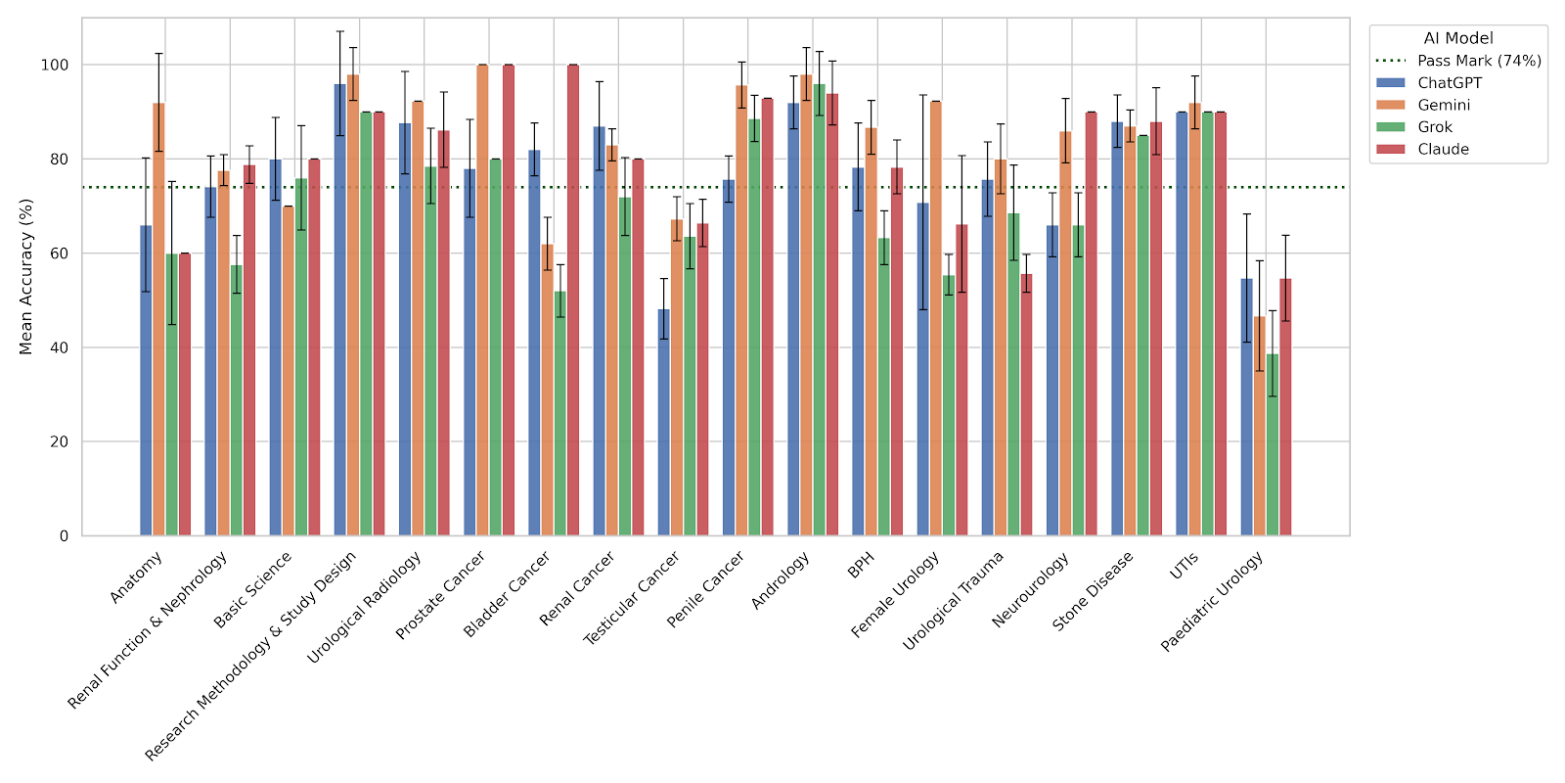
Subspecialty performance heatmap. Comparative accuracy of four LLMs across 18 urological domains and total examination score. Cell values denote the mean accuracy (%) ± SD with colour intensity indicating performance relative to the dataset (dark green for high scores and dark red for low).

The four AI models demonstrated variable performance across urology topics (figures3^5^, table 3). Gemini consistently achieved the highest scores, particularly in anatomy (92.0%), research methodology (98.0%), prostate and penile cancer (100.0% and 95.7%) and andrology (98.0%). ChatGPT and Claude performed strongly in basic science, stone disease and infections, while Grok generally scored lower, especially in female urology (55.4%), bladder cancer (52.0%) and paediatric urology (38.7%). Variability across models was greatest in topics such as bladder cancer, testicular cancer and urological trauma.

**Figure 4:**
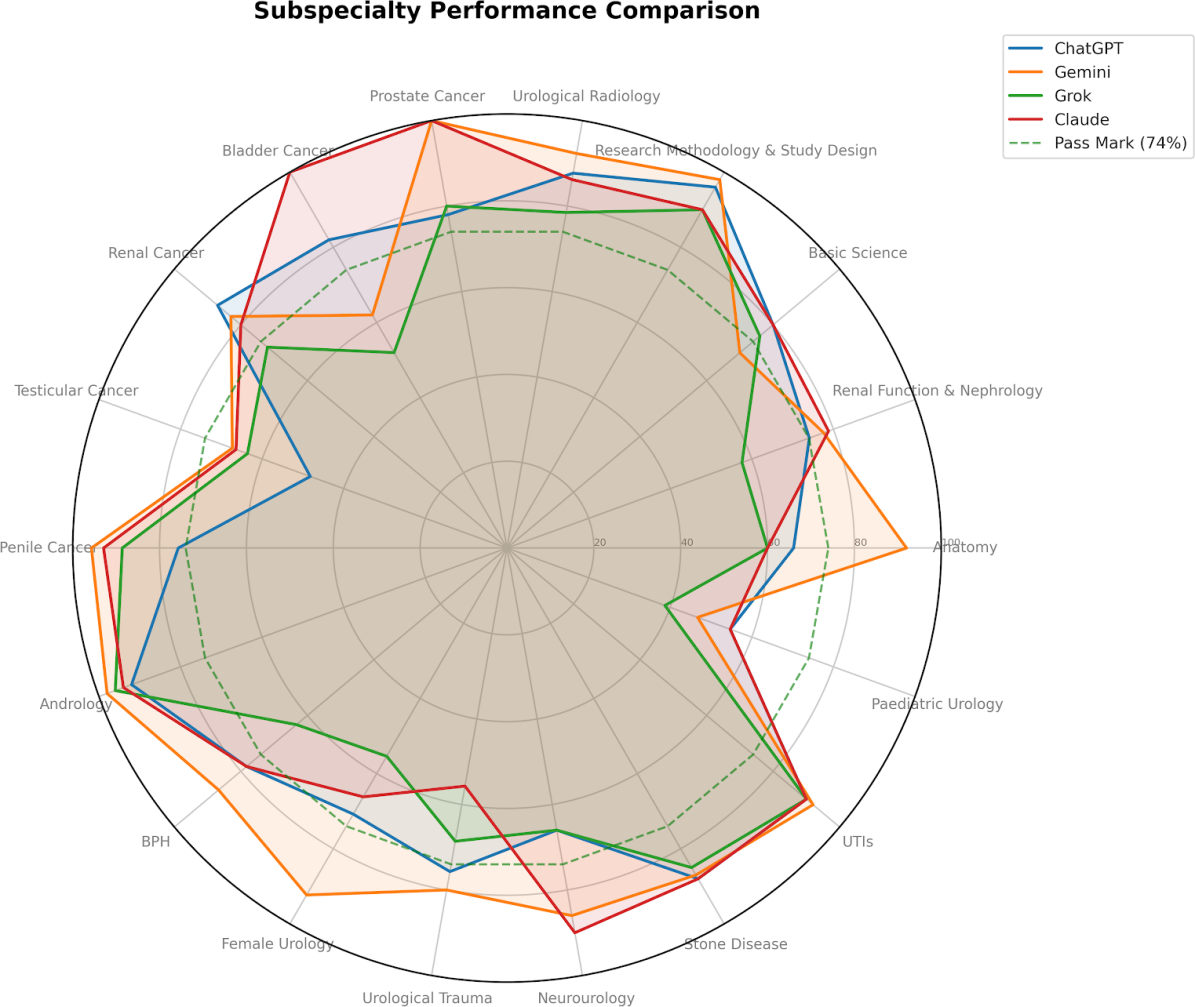
Radar chart comparing accuracy of four AI models across urology subspecialties.

**Figure 5:**
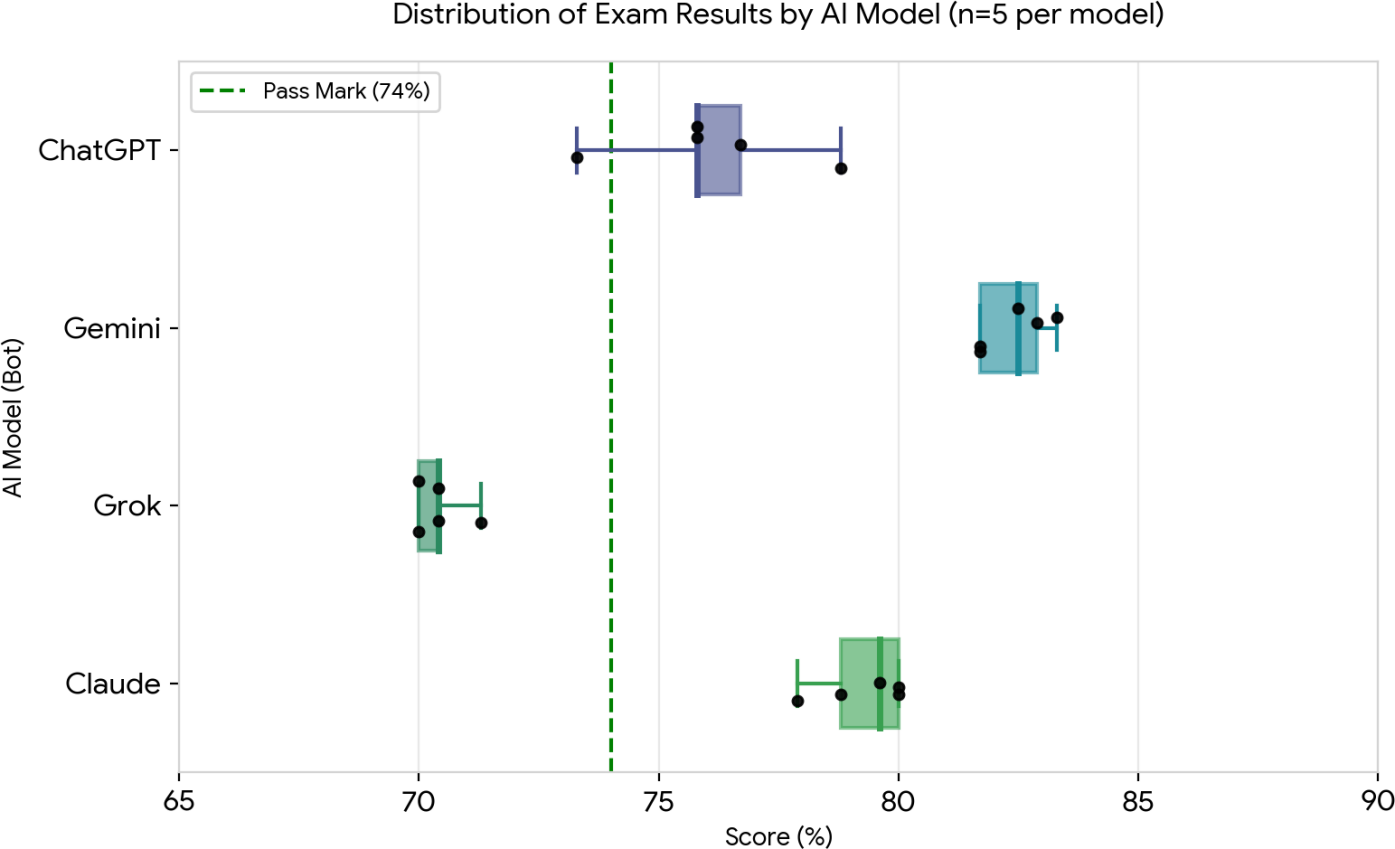
Distribution of overall exam scores for four AI models.

**Table 3:** Performance of AI models across urology topics (percentages±SD)

Topic	ChatGPT (%)	Gemini (%)	Grok (%)	Claude (%)
Anatomy	66.0±14.2	92.0±10.4	60.0±15.2	60.0±0
Renal function and nephrology	74.1±6.5	77.6±3.3	57.6±6.1	78.8±4.0
Basic science	80.0±8.8	70.0±0	76.0±11.1	80.0±0
Research methodology and study designradiology	96.0±11.1	98.0±5.6	90.0±0	90.0±0
Urological Radiology	87.7±10.9	92.3±0	78.5±8.0	86.2±8.0
Prostate cancer	78.0±10.4	100.0±0	80.0±0	100.0±0
Bladder cancer	82.0±5.6	62.0±5.6	52.0±5.6	100.0±0
Renal cancer	87.0±9.4	83.0±3.4	72.0±8.3	80.0±0
Testicular cancer	48.2±6.4	67.3±4.7	63.6±6.9	66.4±5.0
Penile cancer	75.7±4.9	95.7±4.9	88.6±4.9	92.9±0
Andrology	92.0±5.6	98.0±5.6	96.0±6.8	94.0±6.8
Benign prostatic hyperplasia (BPH)	78.3±9.3	86.7±5.7	63.3±5.7	78.3±5.7
Female urology	70.8±22.8	92.3±0	55.4±4.3	66.2±14.5
Urological trauma	75.7±7.9	80.0±7.4	68.6±10.1	55.7±4.0
Neurourology	66.0±6.8	86.0±6.8	66.0±6.8	90.0±0
Stone disease	88.0±5.6	87.0±3.4	85.0±0	88.0±7.1
Upper and lower urinary tract infections	90.0±0	92.0±5.6	90.0±0	90.0±0
Paediatric urology	54.7±13.6	46.7±11.7	38.7±9.1	54.7±9.1
**TOTAL**	**76.1±2.4**	**82.4±0.9**	**70.4±0.6**	**79.3±1.1**

## Discussion

This study evaluated the performance of four contemporary large language models: GPT-5.2 (OpenAI), Gemini 3 Pro (Google DeepMind), Claude Sonnet 4.6 (Anthropic) and Grok 4.1 (xAI) on 240 questions from two simulated FRCS (Urol) Part A examinations. The principal finding is that three of the four models achieved mean scores that exceed the indicative 74% pass threshold: Gemini 3 Pro performed highest (82.4%±0.9%; 95% CI 81.3 to 83.6%), followed by Claude Sonnet 4.6 (79.3%±1.1%; 95% CI 77.9 to 80.6%) and GPT-5.2 (76.1%±2.4%; 95% CI 73.1 to 79.1%). Grok 4.1 achieved the lowest score (70.4%±0.6%; 95% CI 69.6 to 71.2%), falling below this threshold. Notably, Grok’s entire 95% CI lies below 74%, suggesting that its failure to meet the pass standard is unlikely to reflect chance variation. By contrast, GPT-5.2’s confidence interval (73.1–79.1%) straddles the 74% mark, indicating that its pass status would depend on the sitting-specific calibration applied in a live examination.

All four models completed the full 240-question assessment in under 3 minutes, a striking contrast to the 4.5-hour allocation provided to human candidates, demonstrating the computational speed of these systems as rapid knowledge retrieval tools. Considerable variability was observed across subspecialty domains, with all models performing strongly in research methodology (90–98%), andrology (92–98%), upper and lower urinary tract infections (90–92%) and stone disease (85–88%), while collectively demonstrating the weakest performance in paediatric urology (38.7–54.7%) and testicular cancer (48.2–67.3%).

Beyond mean accuracy, domain-level SD revealed marked differences in the consistency of model outputs across repeated runs. The most striking example was ChatGPT’s performance in female urology, where a mean of 70.8% was accompanied by a SD of±22.8%, the largest in the entire dataset, indicating that scores in this domain ranged widely across sessions and cannot be considered stable or reproducible. Similarly, anatomy showed high run-to-run instability for both ChatGPT (±14.2%) and Grok (±15.2%), despite these models achieving only modest mean scores of 66.0% and 60.0%, respectively. Paediatric urology was characterised by substantial variability across all models (SD range ±9.1–13.6%), compounding the already poor mean accuracy observed in this domain. Notably, ChatGPT exhibited a SD of±11.1% in research methodology despite achieving a mean of 96.0%, suggesting that even in areas of apparent strength, output consistency is not guaranteed. By contrast, several models demonstrated a SD of zero in specific domains, including Gemini in female urology, urological radiology and prostate cancer; Claude in anatomy, basic science, bladder cancer and neurourology; and Grok in research methodology and stone disease, indicating fully deterministic and reproducible performance in those areas. This divergence between domains of high variability and those of complete consistency is itself an informative finding, suggesting that LLM output stability is topic-dependent rather than a uniform property of any given model.

Several methodological strengths support confidence in these findings. A standardised simulation framework, including identical prompt delivery, independent sessions with conversational memory disabled and deactivated internet access, minimised instruction-induced variability and ensured comparability across models. The use of a sequential Monte Carlo sampling procedure to construct 95% CIs, with sampling continued until the half-width was less than 2.5% of the point estimate, provided a principled and adaptive approach to quantifying performance uncertainty. Questions were systematically mapped to the Joint Committee on Intercollegiate Examinations' Urology Syllabus Blueprint (2023), ensuring broad and representative domain coverage.^11^ Image-based questions were presented using high-resolution figures extracted from the source question bank, enabling multimodal evaluation consistent with actual examination conditions. However, several limitations must be acknowledged. The question bank was static and proprietary; while it was not publicly available, the possibility of partial overlap with preparatory materials accessible during model pretraining cannot be excluded. Models were evaluated through consumer-facing interfaces rather than controlled API access, introducing potential variability attributable to server load and interface-level processing. With five independent runs per model, statistical precision, though enhanced by Monte Carlo sampling, remains limited, and findings may not generalise beyond the question set evaluated. Crucially, response latency reflects computational inference time rather than deliberate cognitive reasoning, and performance on written multiple-choice questions does not encompass the clinical judgement, operative skill, and professional attributes assessed across the broader FRCS examination structure.

Contextualised within the existing literature, the performance of all four models represents a substantial improvement over earlier evaluations of LLMs on specialist surgical examinations. Desouky *et al* previously reported that ChatGPT-3.5 failed the FRCS Urology examination with a score of 35%, concluding that the model was insufficiently equipped for high-stakes specialist assessment at that time.^5^ The scores observed in the present study, ranging from 70.4% to 82.4%, indicate a transformative improvement across successive model generations, consistent with the trajectory seen in evaluations of LLMs on other postgraduate medical benchmarks, including the USMLE, where frontier models have progressively approached or exceeded passing standards.^16^ The present study extends this literature by directly comparing four contemporaneous frontier models in a single controlled evaluation, an approach that highlights not only the absolute capability of each model but also the meaningful performance differences that exist between them. Gemini 3 Pro’s superior overall accuracy and lowest score variability (SD 0.9%) distinguish it as the most consistent performer in this dataset, while GPT-5.2’s broader CI and higher standard deviation (SD 2.4%) indicate greater run-to-run instability that has practical relevance when these tools are used repeatedly in an educational context.

The pass mark for the FRCS (Urol) Section 1 (Part A) examination is determined at each sitting using a modified Angoff method, whereby subject matter experts estimate the expected performance of a minimally competent candidate and the cut score is adjusted according to the judged difficulty of that specific, non-disclosed paper. The 74% pass threshold applied here was determined by the question bank provider, and therefore, extrapolation to official examination success should be interpreted cautiously, as this study utilised a static question bank and did not reproduce the full modified Angoff process conducted by a large expert standard-setting panel. Notwithstanding this caveat, the domain-level findings offer educationally meaningful insights.

The concentration of weaker performance in paediatric urology and testicular cancer across all four models perhaps reflects the comparatively limited representation of these subspecialties in publicly accessible medical literature and online educational resources relative to topics such as prostate cancer, urinary tract infections or evidence-based methodology, areas in which training corpora are likely densest and most current. The striking inter-model divergence in bladder cancer performance, ranging from 52.0% (Grok) to 100.0% (Claude), illustrates that aggregate scores can obscure important topic-level heterogeneity and that candidates relying on any single model for examination revision risk receiving inconsistent or unreliable guidance in specific domains. This concern is compounded by the within-model variability identified across runs. A trainee using ChatGPT to revise female urology, for example, may receive markedly different answers to the same questions on different occasions, a consequence of the±22.8% SD observed in that domain. Such instability is clinically and educationally significant: it undermines the reliability of the feedback provided, may reinforce incorrect answers on poor-performing runs and makes it difficult for trainees to gauge their true level of knowledge in that area. Conversely, domains in which models achieved a SD of zero, such as Claude in bladder cancer and neurourology or Gemini in female urology and prostate cancer, may represent areas where these tools offer more dependable supplementary support. These findings suggest that LLMs may be best deployed as adjunctive revision tools, capable of providing rapid, broad-coverage knowledge retrieval and self-testing, rather than as authoritative or sole sources of specialist surgical knowledge. Critically, trainees and educators should be aware that the reliability of AI-generated responses is neither uniform across topics nor consistent across models and that high aggregate accuracy does not preclude substantial domain-specific instability. A framework that triangulates AI-generated content against established textbooks, peer-reviewed guidelines and expert-led teaching is therefore advisable before any integration into formal examination preparation.

A further methodological limitation concerns the use of consumer-facing interfaces rather than direct API access for model evaluation. Output variability in LLMs arises from two distinct but related sources that this study design cannot fully disentangle. The first is intrinsic model stochasticity: research has demonstrated that LLMs exhibit meaningful non-determinism even when temperature is set to zero, with instability varying substantially across tasks and across models, and with variability distributions that deviate from normality, complicating standard uncertainty quantification.^17^ The second source is interface-mediated parameter uncertainty. Consumer web interfaces do not expose sampling parameters such as temperature or top_p to the user, precluding the controlled conditions available through direct API access, including fixed temperature and random seed specification. These parameters operate in the background of commercial chat interfaces and cannot be adjusted by the user, a constraint with direct implications for reproducibility in research contexts.^18^ This concern has been echoed in the medical evaluation literature, where a 2025 preprint examining GPT-4o in emergency diagnostic tasks identified temperature as systematically underreported in LLM clinical research, with default settings in commercial deployments frequently unknown to investigators.^19^

It is important to contextualise this limitation appropriately. For the trainee using these tools as an adjunctive revision aid, the primary use case discussed in this paper, the consumer interface is precisely the environment in which they will encounter these models, and the variability characterised here therefore reflects real-world conditions rather than an artificial constraint. In that sense, evaluating models through consumer interfaces is not only pragmatically justified but arguably more ecologically valid for educational purposes than controlled API testing would be. The limitation is principally relevant to researchers seeking to benchmark model performance with precision and reproducibility; however, future technical evaluations should adopt this approach as standard.

Several important questions remain unanswered by this study and merit investigation in future research. First, this evaluation was limited to Part A written multiple-choice questions; performance on Part B written papers, viva examinations and OSCE-style clinical scenarios, which assess applied clinical reasoning, communication and operative decision-making, remains entirely uncharacterised for these models. Second, all four models evaluated represent point-in-time snapshots of rapidly evolving systems; longitudinal assessment across successive model versions will be necessary to determine whether the performance gains observed between GPT-3.5 and current frontier models continue, plateau, or exhibit domain-specific divergence over time. Third, while this study examined performance under standardised conditions, the effect of prompting strategy, including chain-of-thought reasoning, retrieval-augmented generation or domain-specific fine-tuning, on subspecialty accuracy in areas such as paediatric urology and testicular cancer warrants dedicated evaluation. Finally, the integration of AI tools into formal urology training curricula requires not only performance benchmarking but also prospective study of their impact on trainee learning outcomes, self-directed study behaviour and examination success. The overarching aim of such integration must remain the augmentation of human mentorship and structured education, rather than its replacement.

## Conclusion

This study demonstrates that three of four contemporary frontier large language models: Gemini 3 Pro, Claude Sonnet 4.6 and GPT-5.2 achieved scores exceeding the indicative 74% pass threshold for the FRCS (Urol) Part A examination, representing a transformative advance over the 35% reported for ChatGPT-3.5 in a prior evaluation.^5^ Gemini 3 Pro emerged as the strongest and most consistent performer, while Grok 4.1 failed to meet the pass threshold across all runs. However, aggregate accuracy alone provides an incomplete picture: domain-level analysis revealed clinically meaningful variability, with direct implications for educational reliability. These results suggest that frontier LLMs have reached a capability threshold at which they may serve as viable adjunctive tools in urology examination preparation and self-directed learning, provided they are deployed with an awareness of their domain-specific limitations and output inconsistency. They should not be regarded as authoritative knowledge sources, nor as substitutes for structured expert-led training. As LLMs continue to evolve at pace, ongoing evaluation against validated specialist benchmarks will be essential to ensure their safe, effective and evidence-based integration into surgical education.

## Supplementary material

10.1136/bmjopen-2025-108775 online supplemental file 1

## Data Availability

No data are available.
