# Supplementary material for: Performance of large language models (GPT-5.2, Gemini 3 Pro, Claude Sonnet 4.6 and Grok 4.1) on the Fellowship of The Royal College of Surgeons Urology Part A examination
Source: BMJ Open. 2026 May 13;16(5):e108775. doi: 10.1136/bmjopen-2025-108775 (PMC13182482; doi:10.1136/bmjopen-2025-108775)
Supplement: Supplementary file 2 [file bmjopen-2025-108775.reviewer_comments.pdf]

PEER REVIEW HISTORY

BMJ Open publishes all reviews undertaken for accepted manuscripts. Reviewers are asked to complete a checklist review form and are provided with free text boxes to elaborate on their assessment. These free text comments are reproduced below.

ARTICLE DETAILS

Title (Provisional)

The Performance of Large Language Models (GPT-5.2, Gemini 3 Pro, Claude Sonnet 4.6, and Grok 4.1) on the Fellowship of The Royal College of Surgeons Urology Part A Examination

Authors

Kafagi, Abdul Rhaman; Atassi, Nazih; Kafagi, Abdul-Hadi

VERSION 1 - REVIEW

**Reviewer**	**1**
**Name**	**Dack, Kyle**
**Affiliation**	**University of Bristol, MRC Integrative Epidemiology Unit**
**Date**	**11-Sep-2025**
**COI**	**None**

Summary:

This paper addresses an important research question and the findings could be valuable.

However, currently the study is equivalent to a single case study – the LLM was only tested once. LLM are probabilistic, and from one observation we can't tell if the 74.5% score is the average, or if it was under- or over-performing in this particular circumstance. It is common practice in simulation studies, and it is increasingly recommended in LLM evaluations, to perform multiple runs of the same questions, so that variance and uncertainty can be estimated– i.e, the equivalent of increasing the sample size.

Abstract:

Clear and comprehensive, no suggestions.

Introduction:

No suggestions.

Methods:

Is a reference available for the questions which were asked?

Please report the full specific procedure used to test the LLM. E.g. the prompts used, how responses were recorded and evaluated, how time was measured, were how chat sessions were handled, and any other specifics of the practicalities. Given that this uses a publicly available tool, it should be possible to give enough details that other researchers can replicate this study and arrive at similar results.

Please clarify the statistical analysis – software used, and specific variables which were compared and estimated.

My primary concern is that the test was only applied once. ChatGPT uses probability distributions to select responses, which means that there is an element of randomness and variance. If the model is presented with the same question multiple times, it is unlikely to give an identical response. An analogy would be like performing an observational study on 10 participants – the uncertainty can't be modelled and we can't be sure if the result was fluke.

I am aware that this is also an issue with prior epidemiological evaluations of LLM such as the Desouky et al paper. However, this would be an opportunity to improve the methodology and strengthen the novelty of this paper, and could be noted as a limitation of prior papers.

A commonly recommended remedy in LLM literature is to perform repeated runs or tests, each in a new session. The average performance can then be calculated, along with variance and a certainty measure such as 95% CI. This would allow for much greater confidence in the result, and support stronger conclusions.

This is an emerging methodology and I don't see clear statistical guidance on the number of runs which tends to be suitable, but the authors could try 10 repeated runs, check the variance, and continue in further blocks of 10 until a suitable precision is found in the 95% CI. This is somewhat equivalent to sequential fixed-precision Monte Carlo methods, which are a well established practice in simulation studies.

Refs: https://arxiv.org/html/2406.10229v1#S7,
https://www.medrxiv.org/content/10.1101/2025.08.06.25333170v1.full.pdf ,
https://arxiv.org/abs/2407.10457

Results and discussion

I have no suggestions other than the previous stated ones. The reporting is clear and discussion is reasonable, other than the major concern regarding sample size / certainty.

**Reviewer**	**2**
**Name**	**Challacombe, Ben**
**Affiliation**	**Guy's and St Thomas' Hospitals NHS Trust, urology**

**Date**	**08-Feb-2026**
**COI**	**None**

This paper assess the newest Chat GPT 4

Chat GPT v4 sat 2 mock exams of 120 questions each to simulate the FRCS Urol Part A

the chatbot passed the exam at 74.5%

It took 14.5 minutes to complete the exam (compared to 4 hours 30 mins) with 3.6 seconds per question

Answers only were provided rather than explanations

the models was better in foundation and basic science areas rather than clinical domains

It performed worst in female and functional urology areas

Recommendation:

Short compact and punchy paper

well written with good references and grammar

This study shows how far CHAT GPT and other chatbots have evolved and that they can now pass complex medical examinations.

It is likely that the performances will improve with enhanced versions.

I agree that this shows the system could be an important educational tool. Good for revision as long as it shows some explanation. It might change slightly how we assess trainees for professional examinations.

The downsides of AI/LLMs could be expanded on and the implications for wider educational programme and exams, hallucinations and biases.

It would have been nice compare different LLM- large language model systems against each other in this paper eg Gemini or similar

I think this deserves publication as a novel application of the technology and a really impressive result.

VERSION 1 - AUTHOR RESPONSE

Reviewer 1, Comment 1: Single run / output variability (Major)

> ***Comment:*** *This paper addresses an important research question; however, the LLM was only tested once. LLMs are probabilistic, and a single observation cannot characterise whether the score reflects average performance or chance. Repeated runs are recommended to estimate variance and uncertainty, analogous to increasing sample size.*

Response: We thank the reviewer for identifying this as a fundamental methodological concern, and we agree entirely. This was the primary impetus for the substantial revision of the present manuscript. In the revised study, each of the four models underwent five independent examination runs, each conducted in a new session with conversational memory disabled. A sequential Monte Carlo sampling procedure was used to construct 95% confidence intervals, with sampling continued until the half-width of the CI was less than 2.5% of the point estimate. A principled, adaptive approach to uncertainty quantification that avoids reliance on an arbitrarily fixed number of replicates. This approach is described in detail in the Methods (Section 2.4) and directly addresses the reviewer's concern. We have also cited the key methodological references recommended by the reviewer (*see in-text* [12] Shyr et al., [14] 2025; Goldshmidt & Horovicz, 2024). The revised results include mean scores, standard deviations, and 95% CIs for all overall and domain-level outcomes, and the instability identified across repeated runs . We believe this revision substantially strengthens the methodological rigour and novelty of the work relative to prior evaluations in this area, including that of Desouky et al.

Reviewer 1, Comment 2: Reference for questions

Comment: *Is a reference available for the questions which were asked?*

Response: The questions were drawn from a proprietary commercial question bank mapped to the JCIE Urology Syllabus Blueprint (2023). The question bank itself cannot be cited as a publicly accessible source; however, the JCIE Syllabus Blueprint against which questions were mapped is cited in full (Reference 11). We have clarified this in the Methods section (Section 2.1) and acknowledge that the proprietary nature of the question bank is itself a limitation, as partial overlap with preparatory materials accessible during model pretraining cannot be fully excluded - a point noted in the Discussion.

Reviewer 1, Comment 3: Reproducibility of procedure

Comment: *Please report the full specific procedure used to test the LLM, including prompts used, how responses were recorded and evaluated, how time was measured, how chat sessions were handled, and any other specifics necessary for replication.*

Response: A dedicated Simulation Framework subsection (Section 2.3) has been added to the Methods, providing the verbatim standardised prompt delivered at the start of each session, a description of session management (new independent session per run, conversational memory disabled, internet access deactivated), the method by which image-based questions were presented (high-resolution figures extracted from the source question bank), the process for sequential question submission and response recording, and the marking procedure against the official answer key. Response time measurement is described separately in Section 2.5, including the definition of elapsed time, the use of automated timestamps, and derivation of per-question averages across five runs.

Reviewer 1, Comment 4: Statistical analysis clarification

Comment: *Please clarify the statistical analysis - software used, and specific variables compared and estimated.*

Response: The Statistical Analysis subsection (Section 2.4) has been expanded to specify that data were analysed using Stata and tabulated in Microsoft Excel. The mathematical definition of accuracy rate (A) is provided, and the sequential Monte Carlo procedure used to derive 95% CIs is described with reference to the supporting literature. The benchmark threshold (74%, as determined by the question bank provider

using a process analogous to the modified Angoff method) is also defined in this section.

REVIEWER 2: Mr. Ben Challacombe, Guy's and St Thomas' Hospitals NHS Trust

Reviewer 2, Comment 1: Comparison of multiple LLMs

Comment: *It would have been nice to compare different LLM systems against each other in this paper, e.g. Gemini or similar.*

Response: The revised manuscript directly addresses this suggestion. The study now presents a controlled comparative evaluation of four contemporary frontier LLMs: GPT-5.2 (OpenAI), Gemini 3 Pro (Google DeepMind), Claude Sonnet 4.6 (Anthropic), and Grok 4.1 (xAI). Direct head-to-head comparison across all four models, including overall accuracy, subspecialty-level performance, response time, and output consistency, forms the central contribution of the revised paper.

Reviewer 2, Comment 2: Expand on downsides of AI/LLMs

Comment: *The downsides of AI/LLMs could be expanded on, including implications for wider educational programmes and exams, hallucinations, and biases.*

Response: The Discussion has been substantially expanded to address these points. We have added discussion of: (1) within-model output instability and its implications for educational reliability, including the specific concern that trainees may receive inconsistent answers to identical questions across sessions; (2) the limitations of aggregate accuracy as a performance metric when domain-specific instability exists; (3) the distinction between computational response speed and genuine cognitive reasoning; (4) the inability of Part A multiple-choice performance to capture hallucination, clinical bias, or professional judgement; and (5) a framework recommending that AI-generated content be triangulated against established textbooks, peer-reviewed guidelines, and expert-led teaching before integration into formal examination preparation. A dedicated paragraph on output instability and its consequences for trainee self-assessment has been added, and the recommendation that LLMs serve as adjunctive revision aids rather than authoritative sources has been reinforced throughout.

We hope that the revised manuscript, with its extended comparative design, repeated testing methodology, quantified output instability, and expanded discussion, addresses the concerns raised by both reviewers and the editorial office comprehensively. We are grateful for the opportunity to significantly improve this work.

Please do not hesitate to contact us should you require any further information or clarification.

Yours sincerely,

Abdul Rhaman Kafagi, Corresponding author and guarantor

On behalf of all authors

VERSION 2 - REVIEW

**Reviewer**	**1**
**Name**	**Dack, Kyle**
**Affiliation**	**University of Bristol, MRC Integrative Epidemiology Unit**
**Date**	**24-Mar-2026**
**COI**	

The analysis has been significantly expanded: the model is now tested multiple times allowing variance to be quantified, and multiple models were tested and compared.

The methods are clear and replicable and the statistics are reported and interpreted accurately. Thank you to the authors for their responses, this was an interesting paper to read.

Minor comments:

Section 2.1 is missing a subheading name.

Table 1. The statistics are presented to varying decimal places, e.g. mean time is to 1 dp, time per question is to 3 dp, and SD is to 2 dp. It would improve readability to make these all consistent.
